# Imputation Server PGS: an automated approach to calculate polygenic risk scores on imputation servers

**DOI:** 10.1093/nar/gkae331

**Published:** 2024-05-06

**Authors:** Lukas Forer, Daniel Taliun, Jonathon LeFaive, Albert V Smith, Andrew P Boughton, Stefan Coassin, Claudia Lamina, Florian Kronenberg, Christian Fuchsberger, Sebastian Schönherr

**Affiliations:** Institute of Genetic Epidemiology, Medical University of Innsbruck, Innsbruck, Austria; Canada Excellence Research Chair in Genomic Medicine, McGill University, Montreal, Québec, Canada; Department of Human Genetics, Faculty of Medicine and Health Sciences, McGill University, Montréal, Québec, Canada; Department of Biostatistics and the Center for Statistical Genetics, University of Michigan, Ann Arbor, MI 48109, USA; Department of Biostatistics and the Center for Statistical Genetics, University of Michigan, Ann Arbor, MI 48109, USA; Department of Biostatistics and the Center for Statistical Genetics, University of Michigan, Ann Arbor, MI 48109, USA; Institute of Genetic Epidemiology, Medical University of Innsbruck, Innsbruck, Austria; Institute of Genetic Epidemiology, Medical University of Innsbruck, Innsbruck, Austria; Institute of Genetic Epidemiology, Medical University of Innsbruck, Innsbruck, Austria; Institute of Genetic Epidemiology, Medical University of Innsbruck, Innsbruck, Austria; Department of Biostatistics and the Center for Statistical Genetics, University of Michigan, Ann Arbor, MI 48109, USA; Institute for Biomedicine, Eurac Research, Bolzano, Italy; Institute of Genetic Epidemiology, Medical University of Innsbruck, Innsbruck, Austria

## Abstract

Polygenic scores (PGS) enable the prediction of genetic predisposition for a wide range of traits and diseases by calculating the weighted sum of allele dosages for genetic variants associated with the trait or disease in question. Present approaches for calculating PGS from genotypes are often inefficient and labor-intensive, limiting transferability into clinical applications. Here, we present ‘Imputation Server PGS’, an extension of the Michigan Imputation Server designed to automate a standardized calculation of polygenic scores based on imputed genotypes. This extends the widely used Michigan Imputation Server with new functionality, bringing the simplicity and efficiency of modern imputation to the PGS field. The service currently supports over 4489 published polygenic scores from publicly available repositories and provides extensive quality control, including ancestry estimation to report population stratification. An interactive report empowers users to screen and compare thousands of scores in a fast and intuitive way. Imputation Server PGS provides a user-friendly web service, facilitating the application of polygenic scores to a wide range of genetic studies and is freely available at https://imputationserver.sph.umich.edu.

## Graphical Abstract

**Graphical Abstract:**
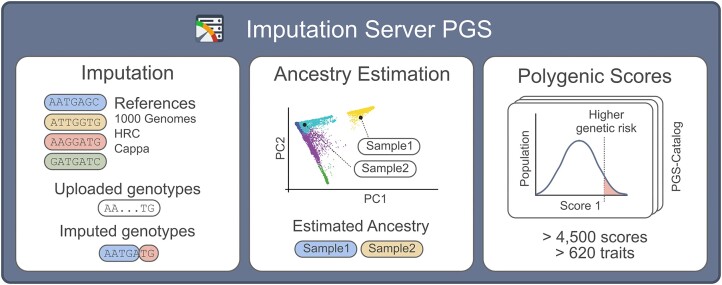

## Introduction

Polygenic scores (PGS), also known as polygenic risk scores (PRS), quantify the impact of genetic variants on complex traits by assigning weights to each variant based on the evidence of statistical associations with the trait discovered through a corresponding genome-wide association study (GWAS) (1). The weights of those variants present in an individual genome are added up to build a single polygenic score. This score reflects the relative genetic risk of a person developing a given disease and helps understanding the genetic architecture of complex traits. Recent studies have shown that PGS will increase the likelihood of identifying patients at high risk (2,3). This may advocate a person for early initiation of lifestyle changes or pharmacological treatment to reduce the overall lifetime risk (4). As GWAS sample sizes increase and polygenic scores become more powerful, they are expected to play a key role in genetic research and to assist in clinical decision-making in the near future (5).

Over the last few years, online repositories such as PGS Catalog (6), Cancer PRSweb (7) and ExPRSweb (8) published thousands of new scores with millions of variants and made them accessible to researchers. Most prominent, the PGS Catalog is an online repository that collects and annotates published scores and currently provides access to over 4489 scores encompassing >650 traits. Other repositories such as Cancer PRSweb and ExPRSweb collect scores for major cancer traits and common health-related exposures like body mass index or alcohol consumption, respectively. Due to the increasing number of scores, PGS calculation can vary significantly in computation time depending on the number of variants included in a score and the implementation of the underlying software program. Thus, a robust and efficient approach to calculate, analyze and validate thousands of polygenic scores is necessary to improve their potential use in clinical settings.

Here, we present Imputation Server PGS, an extension of the Michigan Imputation Server (MIS) (9) to provide PGS calculation as a service. MIS provides a standardized way to perform high-quality genotype imputation using large multi-ethnic haplotype reference panels. By the time of writing, more than 11600 users have imputed >109 million of genomes and the server has successfully delivered >1 Petabyte of analysis results. Genotype imputation allows inferring a large number of genetic variants from a smaller number of measurements (such as array genotypes). Many PGS depend on rare variants, and hence the synergy with imputation facilitates the practical application of PGS to research data. A new PGS calculation module has been integrated into the existing imputation pipeline as an optional feature, enabling the generation of thousands of scores with reasonable overhead in execution time. Either all available scores or a predefined subset of scores (e.g. Cancer or Body Measurement category) can be calculated. Upon completion, the users receive an interactive report of all scores including meta-information, ancestry estimation and population matching for each individual. In addition, the service provides an interface for integrating with other PGS repositories.

## Materials and methods

### PGS calculation

The polygenic risk score for an individual $j$ and a score with $N$ variants is calculated as


\begin{eqnarray*}{{\rm PGS}_j} = \;\mathop \sum \limits_i^N {\beta _i}*{D_{ij}},\end{eqnarray*}

where ${\beta _i}$is the weight of SNP $i$ and ${D_{ij}}$ is the dosage observed for individual $j$ at SNP $i$. The dosages are the results of the genotype imputation and represent the number of copies of the effect allele. They can be between 0 and 2. However, if the dosage represents the non-effect allele, then the weight for that SNP is inverted by switching direction. SNPs where at least one sample has a missing dosage value or where the imputation quality (empirical *r*^2^ imputation value, ER2) is lower than a predefined threshold (default 0.3), are filtered out and will not contribute to the score. This avoids scores of samples with missing genotypes having a constant lower risk compared to samples with complete genotypes (10). Our approach can resolve *strand-flips* automatically by inverting the effect and non-effect alleles. However, in this mode, all palindromic SNPs, where the alleles are G/C or A/T, are filtered out since the direction of the weight cannot be determined. The strand of all other SNPs is detected and resolved automatically. We support *multi-allelic SNPs* if split into multiple records of biallelic SNPs to ensure that only correct weighted dosages are used. Short insertions and deletions (*InDels)* are handled identically to SNPs and are considered when the alleles of the imputed data match the alleles of the score file.

All filtered SNPs are reported, and the coverage of each score is calculated. The coverage is defined as the ratio between the variants found in the imputed datasets (after applying the previously mentioned filters) and the total variants of the score. Based on their coverage, scores are classified into three distinct groups: (i) high (coverage above 75%), (ii) medium (coverage between 25% and 75%) and (iii) low (coverage below 25%).

### Building unified PGS repositories

We automated the process of creating and harmonizing different PGS repositories by implementing a Nextflow (11) pipeline to enable fast and reproducible updates. The pipeline downloads all available scores and metadata from PGS Catalog using the REST API. Next, it merges the weights of all scores into a single repository file by grouping them by variants. This facilitates the creation of an index on variant positions (using Tabix (12)) for efficient extraction of variants in each chunk. Finally, the workflow generates an annotation file for each score, including information such as (a) populations and sample counts from the GWAS used for weight construction, (b) trait details, (c) publication information, (d) and the number of variants. This pipeline provides an automated synchronization mechanism with the PGS Catalog. We are planning to release PGS Catalog updates once a month.

### Ancestry estimation

First, we use LASER to perform principal components analysis (PCA) based on the genotypes of each sample and to place them into a reference PCA space constructed using a set of reference individuals with known ancestries (13). We built the PCA reference coordinates based on 938 samples from the Human Genome Diversity Project (HGDP) (14) and labeled them by the ancestry categories proposed by the GWASCatalog (15), which are also used in the PGS Catalog. The seven super populations are African (AFR), European (EUR), Greater Middle Eastern (GME), East Asian (EAS), South Asian (SAS), Hispanic or Latin American (AMR) and Additional Diverse Ancestries (e.g. Oceania) (OTH).

Next, we implemented a K-Nearest-Neighbor (K-NN) classifier to estimate the population of each uploaded sample similar as proposed by Zhang et al. (16). For each sample, we calculate the Euclidian distance to the reference samples based on the first 10 PCs. It has been shown that the (squared) Euclidean distance correlates with genetic dissimilarity between populations (*F_ST_*) and can be used to find an appropriate clustering of populations (17). Finally, we use the 20 nearest reference samples to determine the population. For that purpose, we weight their population label by the inverse distance so that reference samples that are similar to the unclassified sample have a higher weight than those that are farther away in the PCA space. If a population has more than 75% of the total weighted votes, then we classify the sample with this population label. Otherwise, if no accurate estimation is possible, we label the sample as unclassified. The classifier itself is implemented in Java and is part of our PGS extension.

### Parallelization and integration into Michigan Imputation Server

**Figure 1.**
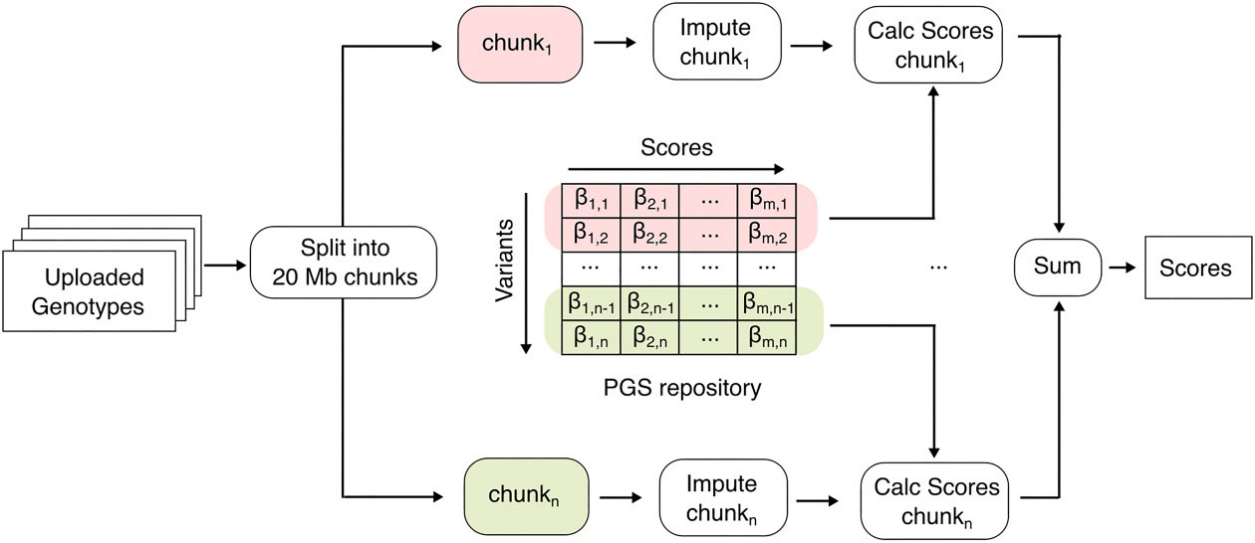
Parallelization workflow. The uploaded genotypes are split into 20 Mb chunks and genotype phasing as well as imputation is performed in parallel. For PGS calculation, only the relevant variants are extracted from the PGS repository using the Tabix index. If a variant is not part of the score, the corresponding weight is set to zero.

Michigan Imputation Server, which relies on the Cloudgene framework (18), splits the uploaded genotypes into 20 Mb chunks and distributes them to all available computing nodes. This allows utilizing large cluster architectures efficiently and process chunks in parallel. In a first step, input validation and quality control (QC) is executed. The web-service verifies the files, reports a summary of input parameters and calculates QC statistics such as reference overlap, variant matches and strand or allele switches. Furthermore, a list of filtered sites and a graphical QC report is returned to the user. If the job passes the QC step, phasing and genotype imputation is executed in parallel. To further take advantage of this parallelization model, we also calculate all selected polygenic scores on each imputed chunk. Then, we calculate the final scores by summing up the sub-scores calculated for each chunk (see Figure 1). After PGS calculation, LASER uses the quality-controlled uploaded genotypes to calculate the principal components (PCs). Similar to the original implementation in the LASER server (13), we perform this calculation in parallel by generating batches consisting of up to 50 samples. Next, the ancestry estimator module reads the PCs for each sample and estimates the population using the reference data from HGDP. This estimated population is then compared with the populations of each score provided by the annotation file of the PGS repository. If the estimated population of a sample is not included in this list, it is excluded from this score. Moreover, all samples that are labeled as unclassified are also excluded (e.g. admixed individuals).

In the last step, the report tool combines the results of the PGS calculation and the information from the ancestry estimator to create an HTML report for each score containing (a) meta-information, (b) interactive distribution plots (c) warning messages about possible population mismatches and excluded samples.

## Results

### Polygenic score calculation on Michigan Imputation Server

**Figure 2.**
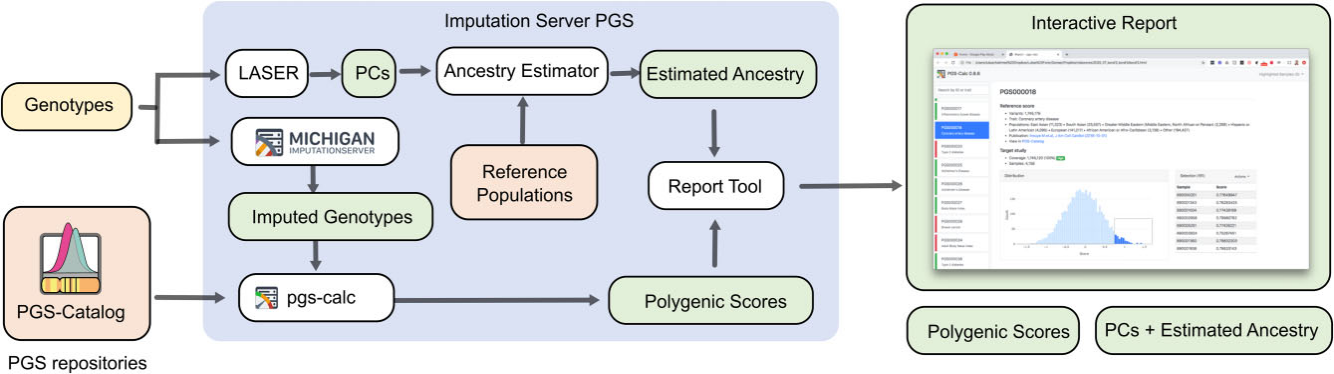
Analysis workflow. First, users upload their data via a web-interface to the Michigan Imputation Server for imputation. Next, the pgs-calc module calculates all selected scores from the PGS repository using the imputed genotypes. Concurrently, LASER is used to calculate the principal components (PCs) and to estimate the ancestry based on a reference population. Finally, the report tool creates an interactive report by merging the scores with the estimated ancestry information and includes distribution plots for each score.

We integrated the PGS calculation and ancestry estimation into the Michigan Imputation Server workflow as an optional step, executed after genotype imputation (see Figure 2). For this purpose, we extended the user interface by a new input block that lists all available PGS repositories and enables users to select scores by trait categories. Users can start the PGS workflow in any web-browser by uploading the genotypes and choosing a reference panel and a PGS repository. Currently, we support 4 reference panels (Haplotype Reference Consortium, 1000 Genomes Phase 1 and Phase 3, CAAPA), 4489 scores and 17 different trait categories (see Supplementary figure S1). After uploading the genotypes, a new imputation job is submitted. Jobs are queued until resources are available to impute the data and to calculate the scores. Users can then download the scores and an interactive report. We provide all calculated scores as a tab-delimited file, which could be loaded into any statistical software for further analysis. In addition, the estimated population and PCs calculated by LASER are also downloadable as text files.

### Security and privacy

For security reasons and to save storage, all uploaded and imputed data are permanently deleted as soon as the process of PGS calculation is finished. The PGS job including all generated results is deleted after 7 days. Furthermore, to monitor jobs and protect any sensitive data, PGS users must register and login to use the service. In case no email address is provided, users can still use the entire functionality of the PGS module. However, if an e-mail address is provided upon registration, users additionally get a notification when the job is completed. The complete interaction with the server is secured with HTTPS. To respect the user's privacy, we limit cookie usage exclusively to login purposes. We do not track any personal information or analyze user activities (see https://imputationserver.readthedocs.io/en/latest/data-sensitivity).

### Interactive report

The report contains a list of all scores, in which each score has a distinct color based on its coverage. Green indicates that the coverage is very high and nearly all SNPs from the score were also found in the imputed dataset. Red indicates that very few SNPs were found and the coverage is therefore low (see Materials and Methods). In addition, the report includes detailed metadata for each score such as the number of variants, the number of well-imputed genotypes and the population used to construct the score. A direct link to PGS Catalog is also available for further investigation (e.g. for getting information about the method that was used to construct the score). Further, the report displays the distribution of the scores of all uploaded samples and can be interactively explored. This allows users to detect samples with either a higher or lower risk immediately.

### Ancestry estimation

Imputation Server PGS includes an overview of all estimated ancestries from the uploaded genotypes and compares them with the populations of the GWAS that was used to create the score. If an uploaded sample with an unsupported population is detected, a warning message is provided, and the sample is excluded from the summary statistics. We validated our ancestry estimation method by comparing the self-reported ancestry from UK-Biobank (UKBB, *n* = 487 726) (19) to our calculated population. Overall, only 0.57% of the UKB samples could not be matched to one of our seven super populations derived from the GWAS Catalog (see Supplementary figure S2). For example, only 0.22% of the self-reported category ‘British’ have been assigned to the ‘Unknown’ category. We compared our ancestry estimation approach to the results presented in Prive et al. (17), where different PCA-based methods have been evaluated (see Supplementary figure S1). Identical to our findings, only about 0.5% could not be matched when applying a nearest-neighbor classifier approach.

**Figure 3.**
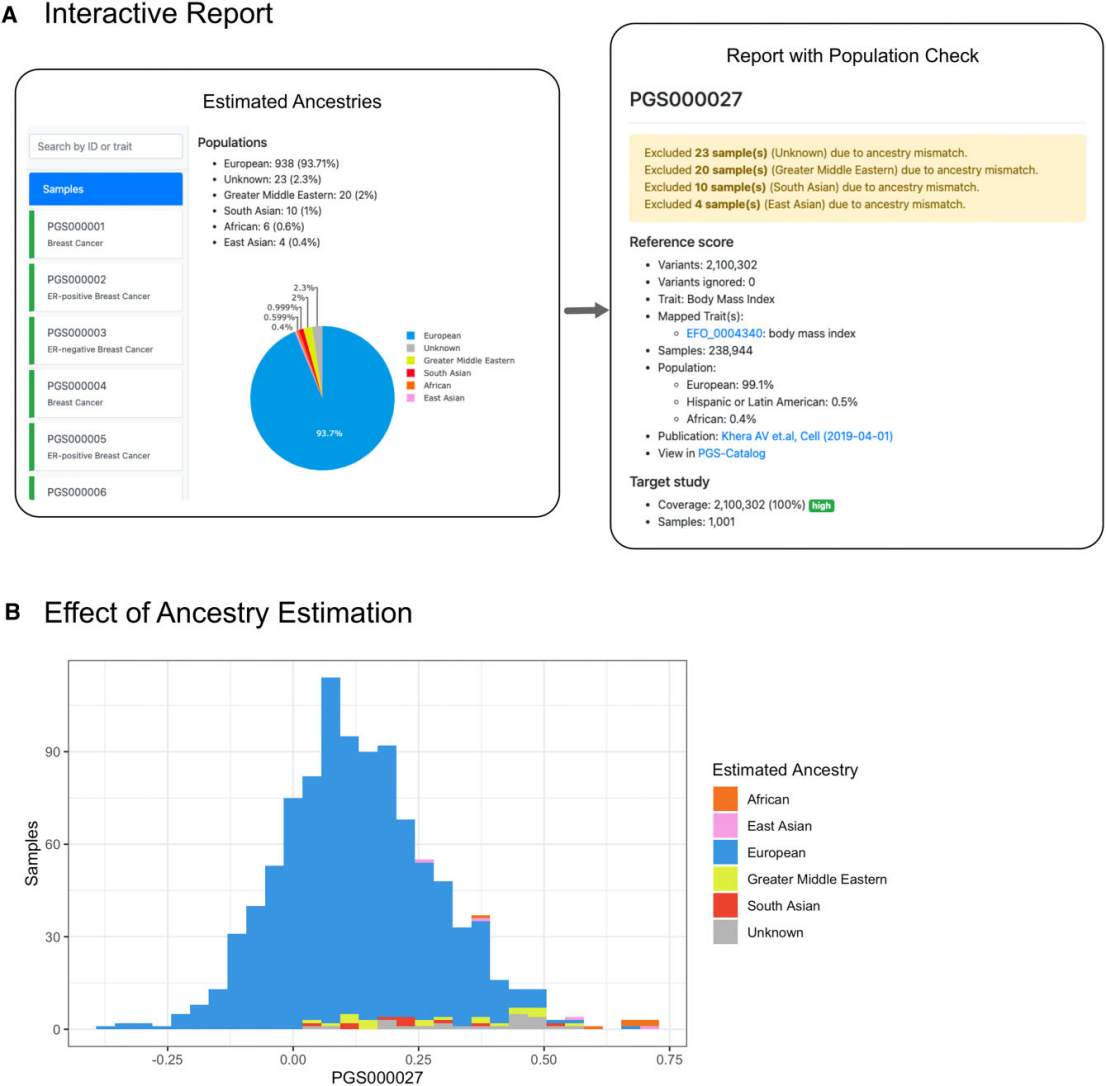
Interactive report and ancestry estimation. (**A**) Imputation Server PGS compares the estimated ancestries with the annotated population for a particular score and highlights all samples from unsupported populations. For example, Score PGS000027 was created mainly on European samples, therefore samples with unknown population, Greater Middles Eastern, South Asian and East Asian are highlighted and excluded from the summary statistics. (**B**) The histogram of all samples (colored by the estimated ancestry) shows that non-European samples are mainly shifted to the right of the distribution. This could lead to miss interpretations in further applications of the score.

We also tested Imputation Server PGS with 1001 in-house samples containing different ancestries in combination with the score PGS000027 (Body mass index) containing over 2 million variants. As this score was constructed mainly based on samples of European genetic ancestry, our approach automatically excludes 57 samples where the estimated genetic ancestry was either Greater Middles Eastern, South Asian, East Asian or not clearly detectable and therefore labeled as unknown population (see Figure 3A). We observed that all excluded non-European samples are mainly shifted to the right of the score distribution relative to Europeans, which could lead to individual risk misclassification if the ancestry is not considered. This highlights the importance of such a feature (see Figure 3B).

### Accuracy and execution time

We performed PGS calculation on Imputation Server PGS for three input datasets with 1000, 2500 and 5000 samples to simulate typical study sizes. For each dataset, we executed imputation and PGS calculations with the following reference panels: 1000G Phase 3 v5 (20), CAAPA (21) and HRC r1.1 2016 (22). Because imputation is such a computationally demanding process, it tends to dominate requirements: as shown in Table 1, the addition of large-scale PGS calculation only marginally extends total job time. Larger sample or reference panel sizes also affect imputation time much more than PGS. For example, for a study with only 1000 samples and a smaller reference panel like the 1000 Genomes reference panel, we observed an overhead of about 20%. As we increase to 5000 samples and the HRC reference panel, the overhead drops to about 15%. To validate our approach, we compared a subset of scores with the results calculated by PLINK2 (23), which is also used by the pgsc_calc command-line tool from PGS Catalog (https://pgsc-calc.readthedocs.io/). Our implementation produces the same results as reported by PLINK2 when using the score sums option.

**Table 1.** Performance evaluation of PGS calculation on Michigan Imputation Server

Reference panel	Samples	Imputation^a^ (min)	PGS-Calculation^a^ (min)	Total^a^ (min)
1000G Phase 3 v5	1000	55.8	13.6	69.3
	2500	122.8	33.6	157.3
	5000	260.7	69.9	329.2
HRC r1.1 2016	1000	79.2	12.7	91.5
	2500	198.4	32.1	230.6
	5000	333.9	59.8	392.5
CAPPA African American Panel	1000	50.6	12.0	62.8
	2500	122.5	31.4	155.1
	5000	237.9	64.8	302.2

^a^Median over all 20 MB chunks.

Experiments with different number of samples and different reference panels show only moderate overhead in the execution time to calculate 4211 scores.

### PGS calculation as a stand-alone tool

The integrated PGS calculation software is also available as a standalone tool called pgs-calc. It reads the imputed dosages from VCF files and uses them in combination with available polygenic weight files to calculate final scores. It supports imputed genotypes in VCF format (e.g. Michigan Imputation Server or TOPMed Imputation Server (24)) and score files from the PGS Catalog. Moreover, the standalone tool enables users to apply their own score files to imputed data and supports them during score development and evaluation. pgs-calc is available at https://github.com/lukfor/pgs-calc.

## Discussion

In recent years, imputation servers have evolved to a key technology in GWAS, especially due to their ease of use and the provision of high-quality imputation. Furthermore, PGS is likely to play a crucial role in genetic research in the future and can contribute to personalized medicine. Here, we presented Imputation Server PGS, a user-friendly web-service to apply thousands of published polygenic risk scores to imputed genotypes. By extending the popular Michigan Imputation Server, we are able to provide PGS calculation within a well-established imputation workflow. The graphical report provides all score meta-data and helps users to understand and screen thousands of scores in an intuitive way. An extensive quality control pipeline is automatically executed to detect and fix strand-flips and to filter out missing SNPs to prevent systematic errors (e.g. lower scores for individuals with missing or wrongly aligned genetic data). For example, Imputation Server PGS enables the calculation of a breast cancer score (PGS000004 (25)), a well-established and validated risk score (26). The results provided by the web-service can then be integrated in external tools like CanRisk (27) to assess the patient's risk (see Supplementary figure S2).

We validated our method against PLINK2 and pgsc_calc and further show that the overhead of the PGS module is minimal higher compared to the imputation-only version of the Michigan Imputation Server. Imputation Server PGS can handle thousands of scores at once by a parallelization on a chunk level instead of a score level. Existing solutions are currently limited either to local executions on the command-line or to web-services like PRSCalc (28), which are designed for direct-to-consumer genetic data, handling only single samples and one score per session (see Table 2). In contrast, software tools such as PRS-CS (29) and PRSice (30) are especially focusing on the creation of novel scores based on GWAS summary statistics. Advantages of a web service for calculating PGS include its accessibility from any device which facilitates usage and collaboration for researchers without deeper knowledge of the underlying computational architecture. Moreover, it requires no in-house computational power or local software installations. However, limitations may arise in terms of customizability and the creation of personalized scores, as well as the need to upload sensitive genetic information.

**Table 2.** Comparison with related software

	Imputation Server PGS	pgsc_calc	PRScalc
Web Service	Yes	No	Yes
File Upload	Yes	No	No
Target	Studies	Studies	Single sample (direct-to-consumer)
Multiple scores	Yes	Yes	No
Multiple samples	Yes	Yes	No
Supported scores	PGS Catalog	PGS Catalog and custom scores	PGS Catalog
Input formats	Genotypes(vcf files)	Imputed Genotypes(vcf, plink1 and plink2 files)	Genotypes (23andMe)
Output formats	csv	csv	json
Html reporting	Yes	Yes	No
Quality control	Yes	No	No
Genotype imputation	Yes	No	No
Ancestry estimation	Yes	Yes	No
Scalability	+++	+++	+
Computational efficiency	+++	+++	+
Ease of use	Graphical	Command-Line	Graphical

A detailed list of key features and functionalities among three among related software tools for PGS calculation.

Since non-Europeans are underrepresented in current GWAS, applying polygenic scores to other ancestries can be critical (31). Admixed or underrepresented populations often have limited representation in GWAS, leading to a lack of specific genetic variants or resulting in allele frequency differences. This limitation can affect the accuracy of PGS derived from multi-ancestry GWAS with predominantly non-admixed populations. Thus, population stratification is a fundamental problem in the analysis of polygenic risk scores and score transferability between populations must be taken into account (32). To address this problem and to provide population-specific information, we include ancestry estimation in our workflow. Samples that mismatch the population of a specific score are automatically flagged and excluded from the summary statistics. Users can download the estimated ancestries and the calculated principal components to include them for further analysis. Our results show that this approach is comparable to other ancestry estimation methods. However, if individuals are distant from all available HGDP sub-populations, then our approach is unable to find a match. This especially affects admixed samples located between clusters, which are then flagged as unknown and excluded. Future work will include the expansion of reference data to include more diverse populations, thereby enhancing coverage of global genome diversity which will further improve the presented clustering approach.

Since not all scores in the PGS Catalog have been validated, it is important to verify its reliability by assessing the underlying GWAS analysis. By providing direct links to the PGS Catalog and the corresponding paper, we encourage users to check if the GWAS is adequately powered, replicated, and if the score has been validated in multiple cohorts and is transferable to the uploaded data (17). In future iterations, we will consider providing more information on PGS accuracy and a rating based on current benchmarking efforts. This enhancement will enable users to get a comprehensive overview of the accuracy and prediction power of the scores provided, helping them choose the appropriate one for their use case. We will also support the upload of custom score files to reduce the overhead associated with using in-house PGS calculation. These new features will improve reproducibility, facilitate benchmarking efforts, and make PGS more accessible. Overall, the new web-service will reduce barriers to using powerful genetic analysis techniques and make standardized PGS calculations available to a wide range of researchers.

## Supplementary Material

gkae331_Supplemental_File

## Data Availability

Imputation Server PGS is available at https://imputationserver.sph.umich.edu. This website is free and open to all users. Upload and analysis of sensitive personal information requires a login. The source code is available at https://github.com/lukfor/pgs-calc (pgs-calc) and https://github.com/genepi/imputationserver. It is also available on Zenodo at https://doi.org/10.5281/zenodo.10973269. The documentation including example data is available at https://imputationserver.readthedocs.io/en/latest/pgs/getting-started/.

## References

[1] Sugrue L.P., Desikan R.S. What are polygenic scores and why are they important?. JAMA. 2019; 321:1820–1821. 30958510 10.1001/jama.2019.3893

[2] Khera A.V., Chaffin M., Aragam K.G., Haas M.E., Roselli C., Choi S.H., Natarajan P., Lander E.S., Lubitz S.A., Ellinor P.T. et al. Genome-wide polygenic scores for common diseases identify individuals with risk equivalent to monogenic mutations. Nat. Genet. 2018; 50:1219–1224. 30104762 10.1038/s41588-018-0183-z PMC6128408

[3] Natarajan P., Peloso G.M., Zekavat S.M., Montasser M., Ganna A., Chaffin M., Khera A.V., Zhou W., Bloom J.M., Engreitz J.M. et al. Deep-coverage whole genome sequences and blood lipids among 16,324 individuals. Nat. Commun. 2018; 9:3391. 30140000 10.1038/s41467-018-05747-8 PMC6107638

[4] Christoffersen M., Tybjaerg-Hansen A. Polygenic risk scores: how much do they add?. Curr. Opin. Lipidol. 2021; 32:157–162. 33900274 10.1097/MOL.0000000000000759

[5] Lewis C.M., Vassos E. Polygenic risk scores: from research tools to clinical instruments. Genome Med. 2020; 12:44. 32423490 10.1186/s13073-020-00742-5 PMC7236300

[6] Lambert S.A., Gil L., Jupp S., Ritchie S.C., Xu Y., Buniello A., McMahon A., Abraham G., Chapman M., Parkinson H. et al. The Polygenic Score Catalog as an open database for reproducibility and systematic evaluation. Nat. Genet. 2021; 53:420–425. 33692568 10.1038/s41588-021-00783-5 PMC11165303

[7] Fritsche L.G., Patil S., Beesley L.J., VandeHaar P., Salvatore M., Ma Y., Peng R.B., Taliun D., Zhou X., Mukherjee B. Cancer PRSweb: an online repository with polygenic risk scores for major cancer traits and their evaluation in two independent biobanks. Am. J. Hum. Genet. 2020; 107:815–836. 32991828 10.1016/j.ajhg.2020.08.025 PMC7675001

[8] Ma Y., Patil S., Zhou X., Mukherjee B., Fritsche L.G. ExPRSweb: an online repository with polygenic risk scores for common health-related exposures. Am. J. Hum. Genet. 2022; 109:1742–1760. 36152628 10.1016/j.ajhg.2022.09.001 PMC9606385

[9] Das S., Forer L., Schonherr S., Sidore C., Locke A.E., Kwong A., Vrieze S.I., Chew E.Y., Levy S., McGue M. et al. Next-generation genotype imputation service and methods. Nat. Genet. 2016; 48:1284–1287. 27571263 10.1038/ng.3656 PMC5157836

[10] Collister J.A., Liu X., Clifton L. Calculating Polygenic Risk Scores (PRS) in UK Biobank: a practical guide for epidemiologists. Front. Genet. 2022; 13:818574. 35251129 10.3389/fgene.2022.818574 PMC8894758

[11] Di Tommaso P., Chatzou M., Floden E.W., Barja P.P., Palumbo E., Notredame C. Nextflow enables reproducible computational workflows. Nat. Biotechnol. 2017; 35:316–319. 28398311 10.1038/nbt.3820

[12] Li H. Tabix: fast retrieval of sequence features from generic TAB-delimited files. Bioinformatics. 2011; 27:718–719. 21208982 10.1093/bioinformatics/btq671 PMC3042176

[13] Taliun D., Chothani S.P., Schonherr S., Forer L., Boehnke M., Abecasis G.R., Wang C. LASER server: ancestry tracing with genotypes or sequence reads. Bioinformatics. 2017; 33:2056–2058. 28200055 10.1093/bioinformatics/btx075 PMC5870850

[14] Li J.Z., Absher D.M., Tang H., Southwick A.M., Casto A.M., Ramachandran S., Cann H.M., Barsh G.S., Feldman M., Cavalli-Sforza L.L. et al. Worldwide human relationships inferred from genome-wide patterns of variation. Science. 2008; 319:1100–1104. 18292342 10.1126/science.1153717

[15] Morales J., Welter D., Bowler E.H., Cerezo M., Harris L.W., McMahon A.C., Hall P., Junkins H.A., Milano A., Hastings E. et al. A standardized framework for representation of ancestry data in genomics studies, with application to the NHGRI-EBI GWAS Catalog. Genome Biol. 2018; 19:21. 29448949 10.1186/s13059-018-1396-2 PMC5815218

[16] Zhang D., Dey R., Lee S. Fast and robust ancestry prediction using principal component analysis. Bioinformatics. 2020; 36:3439–3446. 32196066 10.1093/bioinformatics/btaa152 PMC7267814

[17] Prive F., Aschard H., Carmi S., Folkersen L., Hoggart C., O’Reilly P.F., Vilhjalmsson B.J. Portability of 245 polygenic scores when derived from the UK Biobank and applied to 9 ancestry groups from the same cohort. Am. J. Hum. Genet. 2022; 109:12–23. 34995502 10.1016/j.ajhg.2021.11.008 PMC8764121

[18] Schönherr S., Forer L., Weißensteiner H., Kronenberg F., Specht G., Kloss-Brandstätter A. Cloudgene: a graphical execution platform for MapReduce programs on private and public clouds. BMC Bioinf. 2012; 13:200. 10.1186/1471-2105-13-200 PMC3532373 22888776

[19] Bycroft C., Freeman C., Petkova D., Band G., Elliott L.T., Sharp K., Motyer A., Vukcevic D., Delaneau O., O’Connell J. et al. The UK Biobank resource with deep phenotyping and genomic data. Nature. 2018; 562:203–209. 30305743 10.1038/s41586-018-0579-z PMC6786975

[20] Auton A., Abecasis G.R., Altshuler D.M., Durbin R.M., Abecasis G.R., Bentley D.R., Chakravarti A., Clark A.G., Donnelly P., Eichler E.E. et al. A global reference for human genetic variation. Nature. 2015; 526:68–74. 26432245 10.1038/nature15393 PMC4750478

[21] Mathias R.A., Taub M.A., Gignoux C.R., Fu W., Musharoff S., O’Connor T.D., Vergara C., Torgerson D.G., Pino-Yanes M., Shringarpure S.S. et al. A continuum of admixture in the Western Hemisphere revealed by the African Diaspora genome. Nat. Commun. 2016; 7:12522. 27725671 10.1038/ncomms12522 PMC5062574

[22] McCarthy S., Das S., Kretzschmar W., Delaneau O., Wood A.R., Teumer A., Kang H.M., Fuchsberger C., Danecek P., Sharp K. et al. A reference panel of 64,976 haplotypes for genotype imputation. Nat. Genet. 2016; 48:1279–1283. 27548312 10.1038/ng.3643 PMC5388176

[23] Chang C.C., Chow C.C., Tellier L.C., Vattikuti S., Purcell S.M., Lee J.J. Second-generation PLINK: rising to the challenge of larger and richer datasets. Gigascience. 2015; 4:7. 25722852 10.1186/s13742-015-0047-8 PMC4342193

[24] Taliun D., Harris D.N., Kessler M.D., Carlson J., Szpiech Z.A., Torres R., Taliun S.A.G., Corvelo A., Gogarten S.M., Kang H.M. et al. Sequencing of 53,831 diverse genomes from the NHLBI TOPMed Program. Nature. 2021; 590:290–299. 33568819 10.1038/s41586-021-03205-y PMC7875770

[25] Mavaddat N., Michailidou K., Dennis J., Lush M., Fachal L., Lee A., Tyrer J.P., Chen T.H., Wang Q., Bolla M.K. et al. Polygenic risk scores for prediction of breast cancer and breast cancer subtypes. Am. J. Hum. Genet. 2019; 104:21–34. 30554720 10.1016/j.ajhg.2018.11.002 PMC6323553

[26] Lakeman I.M.M., Rodriguez-Girondo M.D.M., Lee A., Celosse N., Braspenning M.E., van Engelen K., van de Beek I., van der Hout A.H., Gomez Garcia E.B., Mensenkamp A.R. et al. Clinical applicability of the Polygenic Risk Score for breast cancer risk prediction in familial cases. J. Med. Genet. 2023; 60:327–336. 36137616 10.1136/jmg-2022-108502

[27] Carver T., Hartley S., Lee A., Cunningham A.P., Archer S., Babb de Villiers C., Roberts J., Ruston R., Walter F.M., Tischkowitz M. et al. CanRisk Tool - a web interface for the prediction of breast and ovarian cancer risk and the likelihood of carrying genetic pathogenic variants. Cancer Epidemiol. Biomarkers Prev. 2021; 30:469–473. 33335023 10.1158/1055-9965.EPI-20-1319 PMC7611188

[28] Sandoval L., Jafri S., Balasubramanian J.B., Bhawsar P., Edelson J.L., Martins Y., Maass W., Chanock S.J., Garcia-Closas M., Almeida J.S. PRScalc, a privacy-preserving calculation of raw polygenic risk scores from direct-to-consumer genomics data. Bioinform. Adv. 2023; 3:vbad145. 37868335 10.1093/bioadv/vbad145 PMC10589913

[29] Ge T., Chen C.Y., Ni Y., Feng Y.A., Smoller J.W. Polygenic prediction via Bayesian regression and continuous shrinkage priors. Nat. Commun. 2019; 10:1776. 30992449 10.1038/s41467-019-09718-5 PMC6467998

[30] Choi S.W., O’Reilly P.F PRSice-2: polygenic Risk Score software for biobank-scale data. Gigascience. 2019; 8:giz082. 31307061 10.1093/gigascience/giz082 PMC6629542

[31] Martin A.R., Kanai M., Kamatani Y., Okada Y., Neale B.M., Daly M.J. Clinical use of current polygenic risk scores may exacerbate health disparities. Nat. Genet. 2019; 51:584–591. 30926966 10.1038/s41588-019-0379-x PMC6563838

[32] Kurniansyah N., Goodman M.O., Kelly T.N., Elfassy T., Wiggins K.L., Bis J.C., Guo X., Palmas W., Taylor K.D., Lin H.J. et al. A multi-ethnic polygenic risk score is associated with hypertension prevalence and progression throughout adulthood. Nat. Commun. 2022; 13:3549. 35729114 10.1038/s41467-022-31080-2 PMC9213527

