# Supplementary material for: Imputation Server PGS: an automated approach to calculate polygenic risk scores on imputation servers
Source: Nucleic Acids Res. 2024 May 6;52(W1):W70–7. doi: 10.1093/nar/gkae331 (PMC11223871; doi:10.1093/nar/gkae331)

Supplemental Material

Imputation Server PGS: An automated approach to calculate polygenic risk scores on imputation servers

Lukas Forer[1], Daniel Taliun[2,3], Jonathon LeFaive[4], Albert V. Smith[4], Andrew P. Boughton[4], Stefan Coassin[1], Claudia Lamina[1], Florian Kronenberg[1], Christian Fuchsberger[1,4,5] and Sebastian Schönherr[1]

1. Institute of Genetic Epidemiology, Medical University of Innsbruck, Innsbruck, Austria
2. Canada Excellence Research Chair in Genomic Medicine, McGill University, Montreal, Québec, Canada.
3. Department of Human Genetics, Faculty of Medicine and Health Sciences, McGill University, Montréal, Québec, Canada
4. Department of Biostatistics and the Center for Statistical Genetics, University of Michigan, Ann Arbor, MI 48109
5. Institute for Biomedicine, Eurac Research, Bolzano, Italy

<u>Corresponding Author:</u>

Sebastian Schönherr, PhD
sebastian.schoenherr@i-med.ac.at
Institute of Genetic Epidemiology
Medical University of Innsbruck
Schöpfstraße 41
6020 Innsbruck, Austria

TABLES

Table S1: Summary of the Polygenic Score (PGS) Catalog entries categorized by various biological processes and traits. A diverse range of traits is encompassed. Currently, a total of 4,489 scores are supported (release March. 18, 2024).

Repository	Trait	Scores
PGS Catalog*	Biological process	39
	Body measurement	257
	Cancer	659
	Cardiovascular disease	266
	Cardiovascular measurement	142
	Digestive system disorder	350
	Hematological measurement	342
	Immune system disorder	203
	Inflammatory measurement	46
	Lipid or lipoprotein measurement	339
	Liver enzyme measurement	28
	Metabolic disorder	223
	Neurological disorder	239
	Other disease	260
	Other measurement	1,483
	Other trait	190
	Sex-specific PGS	18

* Release: March. 18, 2024 (4,489 scores)

Table S2: Self-reported ancestry of UKBB and the predicted populations by our method. The left rows are the self-reported ancestry from UKBB (n=487,726, field 21000) and the columns are the predicted population from Imputation Server PGS. Only 0.57% could not be matched to one of our seven super populations derived from the GWAS Catalog: African (AFR), European (EUR), Greater Middle Eastern (GME), East Asian (EAS), South Asian (SAS), Hispanic or Latin American (AMR) and Additional Diverse Ancestries (e.g. Oceania) (OTH).

	AFR	AMR	EAS	EUR	GME	OTH	SAS	Unknown
**British**	2		5	426,273	3,325		423	975
**Irish**				12,714	25		9	6
**White**	1	1	1	499	30		1	12
**Other White**		10		12,560	2414		165	667
**Indian**			2	12	1		5,695	6
**Pakistani**				2			1,739	7
**Bangladeshi**			1				220	
**Chinese**			1,481	1			22	
**Other Asian**	1		359	29	140		1,147	71
**Caribbean**	3,926				19		36	316
**African**	2,970			1	71		2	160
**Other Black**	86				7		6	19
**Asian or Asian British**			2	1	3		35	1
**Black or Black British**	22			2	1			1
**White and Black Caribbean**	30			7	334		18	208
**White and Black African**	11			3	136		16	236
**White and Asian**			9	129	27		548	89
**Unknown**	904	97	660	1,964	1,495	1	1,385	678

FIGURES

Figure S1: First eight PCs from UKBB (Field 22009) and populations predicted by Imputation Server PGS. The predicted population from 487,726 samples from UKBB: African (AFR), European (EUR), Greater Middle Eastern (GME), East Asian (EAS), South Asian (SAS), Hispanic or Latin American (AMR) and Additional Diverse Ancestries (e.g. Oceania) (OTH).

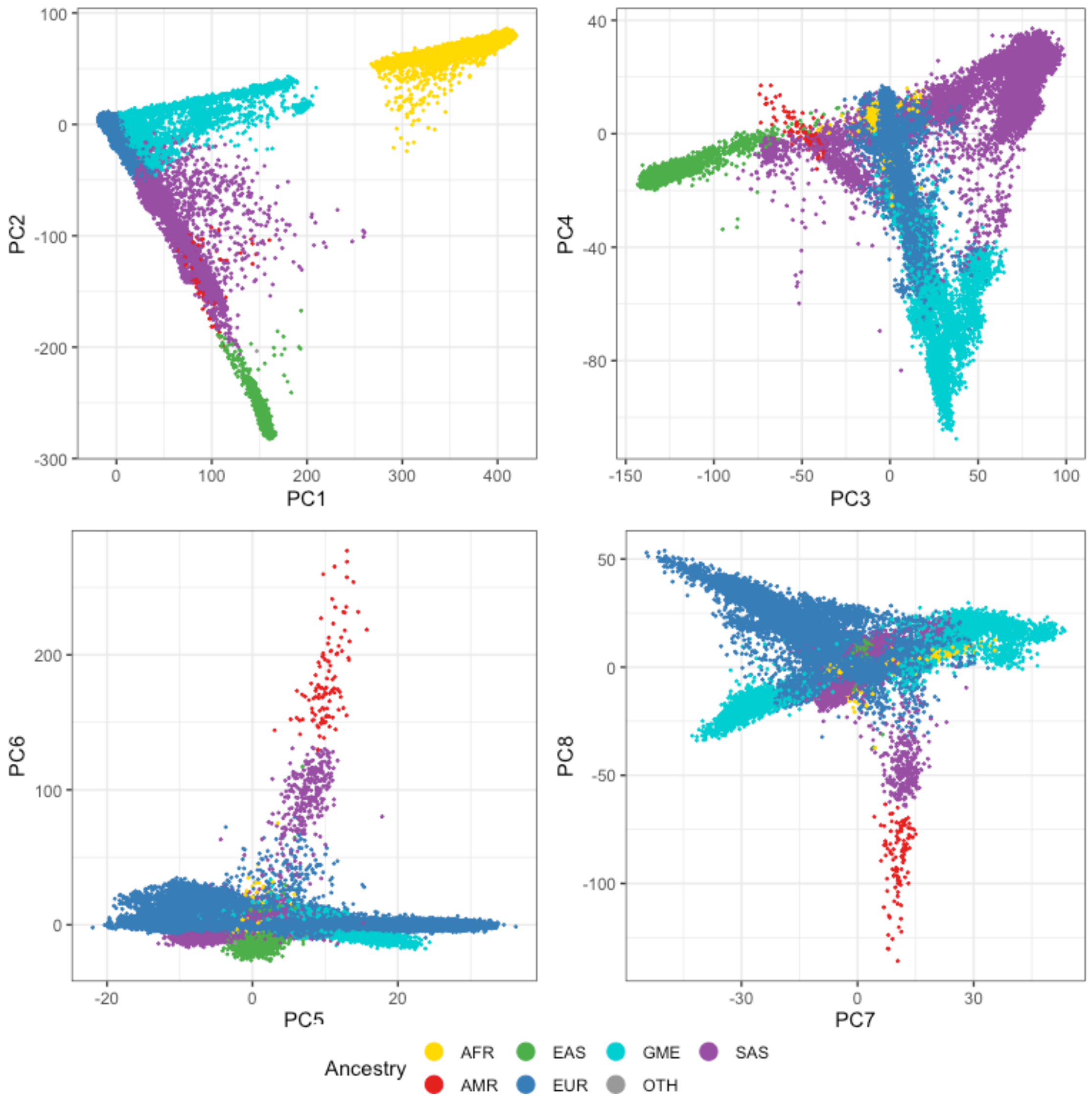

Figure S2: Use case of Imputation Server PGS and breast cancer. Imputation Server PGS enables users to upload genotyping data from patients. Following quality control, genotype imputation, and ancestry estimation, the selected polygenic risk scores are calculated. Score PGS000004 can then be utilized in external tools such as CanRisk to assess the patient's risk.

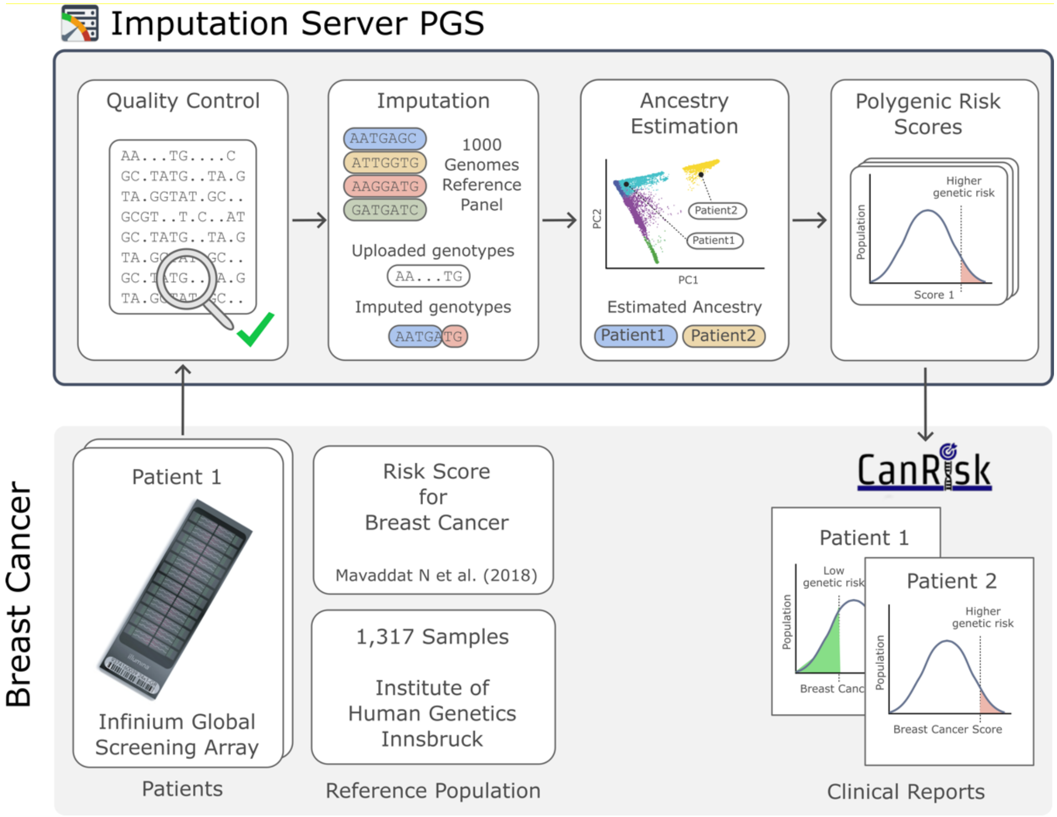

Supplement: gkae331_Supplemental_File [file gkae331_supplemental_file.pdf]
